# Morphologic and Molecular Identification of Human Ocular Infection Caused by *Pelecitus* Nematodes, Thailand

**DOI:** 10.3201/eid3009.231692

**Published:** 2024-09

**Authors:** Ploysai Rujkorakarn, Pukkapol Suvannachart, Samadhi Patamatamkul, Tongjit Thanchomnang, Pairot Pramual, Weerachai Saijuntha, Wanchai Maleewong, Shigehiko Uni

**Affiliations:** Prince of Songkla University, Songkhla, Thailand (P. Rujkorakarn);; Mahasarakham University, Maha Sarakham, Thailand (P. Rujkorakarn, P. Suvannachart, S. Patamatamkul, T. Thanchomnang, P. Pramual, W. Saijuntha);; Khon Kaen University, Khon Kaen, Thailand (P. Suvannachart, W. Maleewong);; Universiti Malaya, Kuala Lumpur, Malaysia (S. Uni); Kobe Women’s University, Kobe, Japan (S. Uni)

**Keywords:** ocular parasitosis, parasites, zoonoses, *Pelecitus*, nematodes, filariae, postdeirid, preesophageal cuticular ring, arboviruses, helminths, Thailand

## Abstract

Nematodes of the Onchocercidae family, such as *Pelecitus* spp., are filarial parasites of medical and veterinary importance. Although infections are widely distributed among avian species, only 2 cases of human *Pelecitus* ocular infection, both in South America, have been reported. We describe a 61-year-old man in northeast Thailand diagnosed with an ocular infection. Morphologic characteristics suggested the causative agent was a female *Pelecitus* nematode: coiled body, rounded anterior and posterior extremities, a distinct preesophageal cuticular ring, lateral alae, a postdeirid, and a protuberant vulva. Sequences of the 12S rDNA gene indicated 95%–96% identity and *cox*1 gene 92%–96% identity with published *P. copsychi* sequences. P-distance for *cox*1 sequences between the causative agent and *P. copsychi* was 6.71%. Phylogenetic trees of 12S rDNA and *cox*1 genes indicated the species differed from but is closely associated with *P. copsychi*. Healthcare providers should be aware of the threat of ocular infection from *Pelecitus* spp. nematodes*.*

Ocular parasitosis is relatively rare, and causative agents vary by geographic area (*1*). Manifestations vary according to the parasite’s location. A live parasite in the anterior chamber of the eye can lead to anterior uveitis or secondary glaucoma. Various parasites, such as representatives of the genera *Gnathostoma*, *Onchocerca*, and *Angiostrongylus*, have been reported in the literature to cause similar conditions (*1*). In Southeast Asia, ocular gnathostomiasis and angiostrongyliasis often manifest with a live parasite in the anterior chamber (*2*,*3*).

The nematode genus *Pelecitus* belongs to the Onchocercidae family, which includes filariae of medical and veterinary importance. Among the 21 species of *Pelecitus* nematodes, 18 are found in birds and 3 in mammals (*4*,*5*), most distributed in Africa and South America (*4*). Birds serve as definitive hosts or reservoirs. *Pelecitus* spp. nematodes are transmitted by blood-sucking arthropods, such as mosquitoes, chewing lice, and tabanids (*6*). 

Within the Indomalayan realm, *P. ceylonensis*, *P. galli*, and *P. copsychi* nematodes have been identified in animal hosts in Sri Lanka and Malaysia (*5*,*7*,*8*). In humans, 2 cases of *Pelecitus* infection have been discovered in Colombia and Brazil (*9*,*10*). However, in both reports, the parasites were identified on the basis of morphologic characteristics only. 

In this study, we identified the causative agent of intraocular infection in a patient outside South America as a nematode species of the genus *Pelecitus*. We subsequently corroborated the preliminary identification based on morphologic characteristics using molecular studies of the mitochondrial 12S ribosomal RNA and the cytochrome *c* oxidase subunit 1 (*cox*1) genes (*11*,*12*). This study provides a morphologic description and details concerning the phylogenetic position of the *Pelecitus* sp. nematode identified in this article. Our case report was approved by the ethics committee of Mahasarakham University (approval no. 181-200/2023). 

## Methods

### Case Report

An otherwise healthy 61-year-old man in Thailand sought treatment for gradually increasing eye pain, redness, light sensitivity, and slight vision loss in his left eye. Symptoms persisted for ≈1 month. He was a farmer in northeastern Thailand and had not traveled outside the country. He regularly consumed traditional dishes containing raw and partially cooked ingredients, including beef and fish. He was diagnosed with an intraocular parasite at a private clinic, where staff performed an Nd:YAG (neodymium-doped yttrium aluminum garnet) laser procedure to immobilize the parasite before the patient was referred to the hospital. We postulate that the infection in this patient might have resulted from a bite from a hematophagous arthropod.

**Figure 1:**
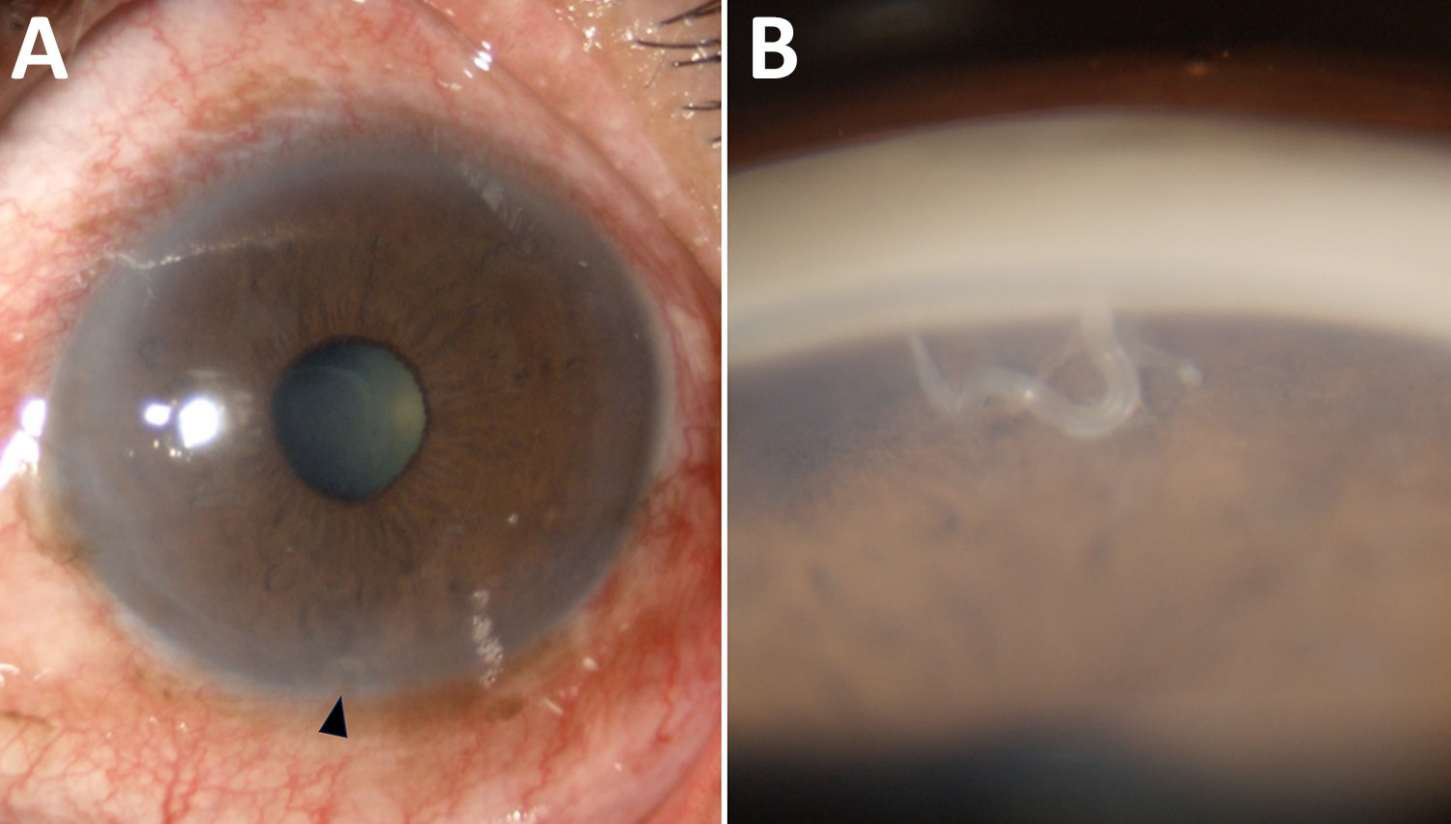
Slit-lamp examination of the left eye of a 61-year-old man in Thailand with ocular parasitosis. A) Ciliary injection and a live parasite in the inferior part of the anterior chamber (arrowhead). B) Gonioscopic view revealing a parasite in the inferior anterior chamber angle, lodged in the iris fibers. Precise magnification levels are not available.

Upon examination, the patient had best-corrected visual acuity of 20/20 in the right eye and 20/40 in the left eye. Intraocular pressure measurements were 18 mm Hg in the right eye and 14 mm Hg in the left. The right eye examination was unremarkable, but the left eye showed ciliary injection (Figure 1, panel A) and grade 1+ anterior chamber cells. A live, moving parasite was observed in the inferior anterior chamber, more distinctly during gonioscopic examination (Figure 1, panel B). The posterior segment was within reference limits. Complete blood count results showed leukocytosis of 12,640 cells/mm^3^ and eosinophilia of 5.5% (absolute eosinophil count 695.2 cells/mm^3^). Fecal examination did not reveal parasites or eggs. 

We performed urgent surgical removal to prevent posterior or extraocular migration. After the operation, we treated the patient with prednisolone acetate 1% eye drops (every 2 h), moxifloxacin 0.5% eye drops (4×/d), and oral albendazole (400 mg 2×/d for 5 d). At 1-month follow-up, the patient returned with a reduction in pain and redness. The best-corrected visual acuity was 20/30 and the intraocular pressure 10 mm Hg in the left eye. We found no active inflammation upon slit-lamp examination. Prednisolone acetate 1% eye drops were gradually tapered. At the 1-year follow-up, the patient was doing well without recurrence of ocular symptoms. We did not perform a blood test at follow-up. 

### Morphologic Identification of the Parasite

We fixed the causative agent in 70% ethanol and subsequently cleared it in lactophenol (R & M Chemicals, https://www.evergreensel.com.my) for morphologic examination under a compound microscope equipped with an Olympus U-DA camera lucida (https://www.olympus-global.com). We extracted DNA from the caudal part of the causative agent (0.2 mm) using the Nucleospin Tissue kit (Macherey-Nagel, https://www.mn-net.com) according to manufacturer instructions. We performed a conventional PCR to amplify the targeted mitochondrial 12S rRNA and *cox*1 genes, according to instructions for specific primers and PCR conditions described elsewhere (*13*–*15*). Afterward, we sequenced the PCR products using the Applied Biosystems DNA Analyzer (ThermoFisher, https://www.thermofisher.com). We compared the 12S rDNA and *cox*1 sequences with public sequences in the GenBank database by using BLASTn (https://blast.ncbi.nlm.nih.gov). 

We performed multiple sequence alignment of DNA sequences from the 12S rRNA and *cox*1 genes with ClustalW (http://www.clustal.org) using MEGA-X (https://www.megasoftware.net). We constructed phylogenetic trees using the maximum-likelihood method with 1,000 bootstrap resamplings as implemented in MEGA-X (*16*). We used publicly available sequences from different nematode species for comparison. We selected the Hasegawa–Kishino–Yano model as a suitable substitution model (*17*).

## Results 

### Morphologic Identification 

**Figure 2:**
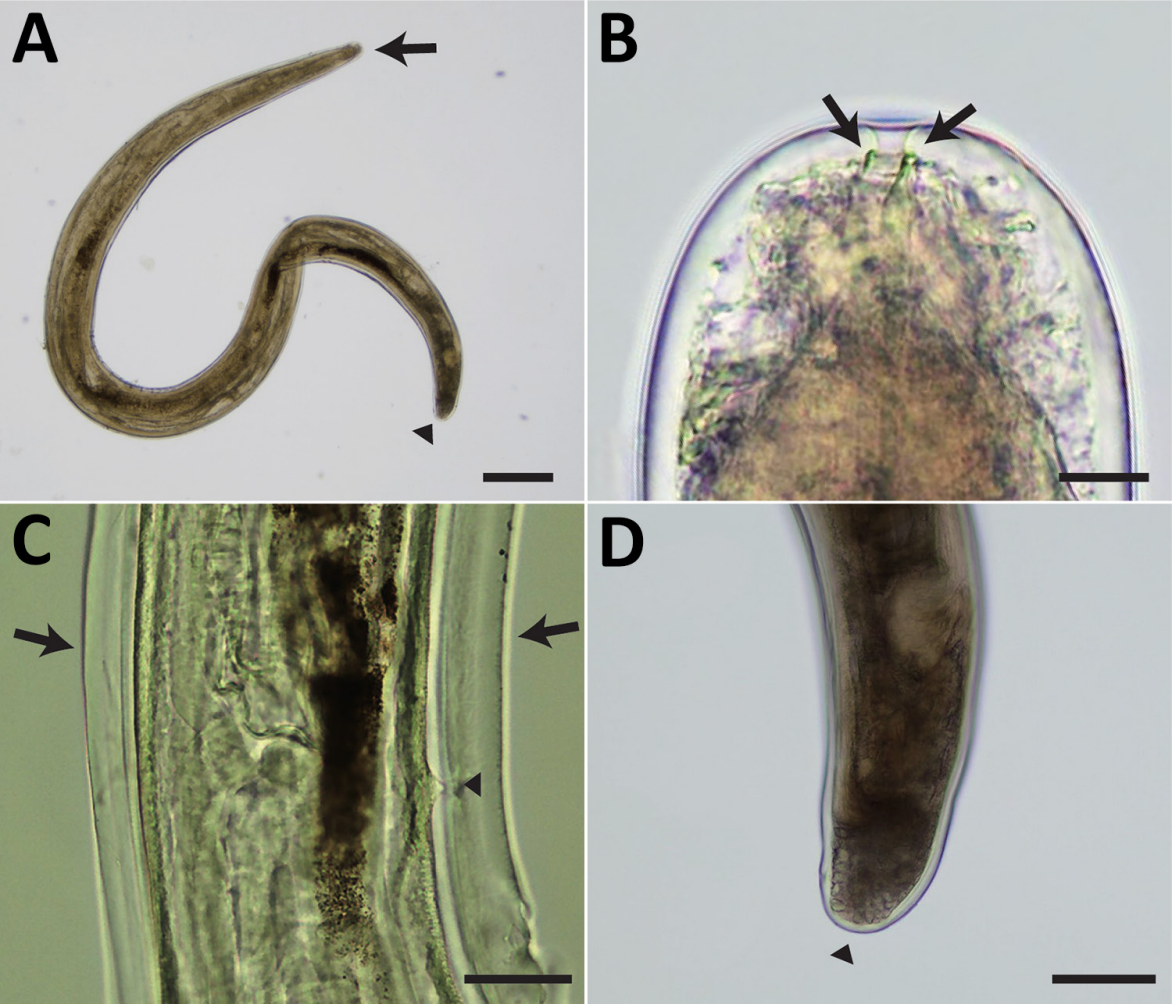
Light micrographs of *Pelecitus* sp. nematode isolated from the left eye of a 61-year-old man in Thailand. A) Curled female, 3.5 mm in length and 198 µm in width. Head (arrow) and tail (arrowhead) are indicated. Scale bar = 250 μm. B) Rounded anterior extremity with preesophageal cuticular ring (arrows). Scale bar = 10 μm. C) Postdeirid (arrowhead) at the posterior left side and lateral alae (arrows) at the posterior part. Scale bar = 50 μm. D) Rounded posterior extremity (arrowhead). Scale bar = 100 μm.

The surgically removed female nematode had a coiled body, 3.5 mm long and 198 μm wide at the midbody (Figure 2, panel A). The head was bluntly rounded and the preesophageal cuticular ring (6.3 µm wide × 2.5 µm high) was distinct (Figure 2, panel B). Labial and cephalic papillae, arranged in 4 submedian pairs, were not markedly protuberant. Amphids were small, lateral, and not salient. A slight neck was found 69 µm from the anterior extremity. The nerve ring was situated 138 µm from the anterior extremity. The esophagus was 608 µm long, and its diameter slightly widened in the posterior half. The vulva was located at the level of the posterior half of the esophagus, 395 µm from the anterior extremity. No microfilariae were present in the uteri. A postdeirid was found on the left side, 300 µm from the caudal extremity (Figure 2, panel C). Lateral alae were present, broadening toward the posterior extremity. At the level of the postdeirid, the ala was 28 µm wide. The intestine was filled with brown pigments. The cuticle was thick, 5–10 µm wide, at the postdeirid level. The tail extremity was rounded (Figure 2, panel D). We deposited the specimen (MNHN-114YT) in the Muséum National d’Histoire Naturelle, Paris, France (https://www.mnhn.fr). 

### Molecular Analyses

**Figure 3:**
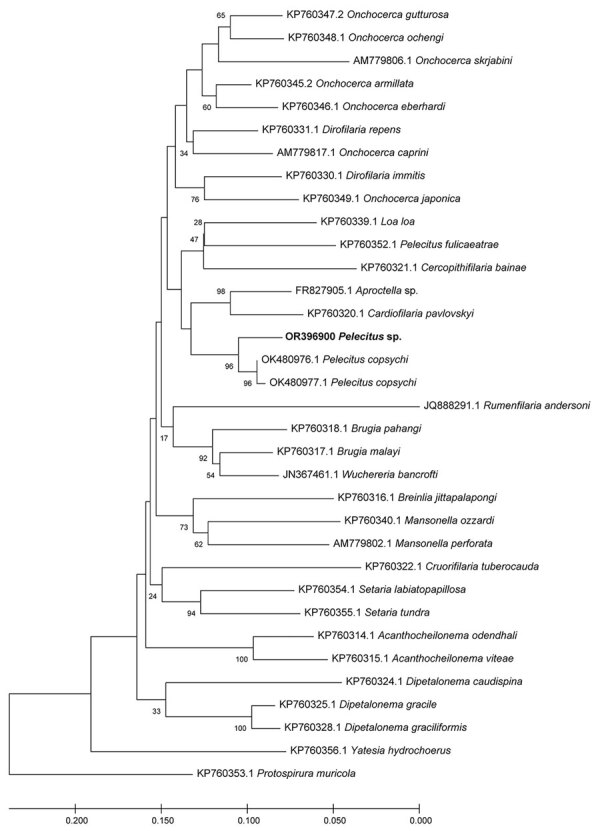
Maximum-likelihood reconstruction of phylogeny on the basis of 12S rDNA sequences of a *Pelecitus* sp. nematode isolated from the left eye of a 61-year-old man in Thailand (bold text) and reference sequences from GenBank. Bootstrap scores (percentages of 1,000 replications) are presented for each node. GenBank accession numbers are shown. Scale bar refers to a phylogenetic distance of 0.05 nucleotide substitutions per site.

**Figure 4:**
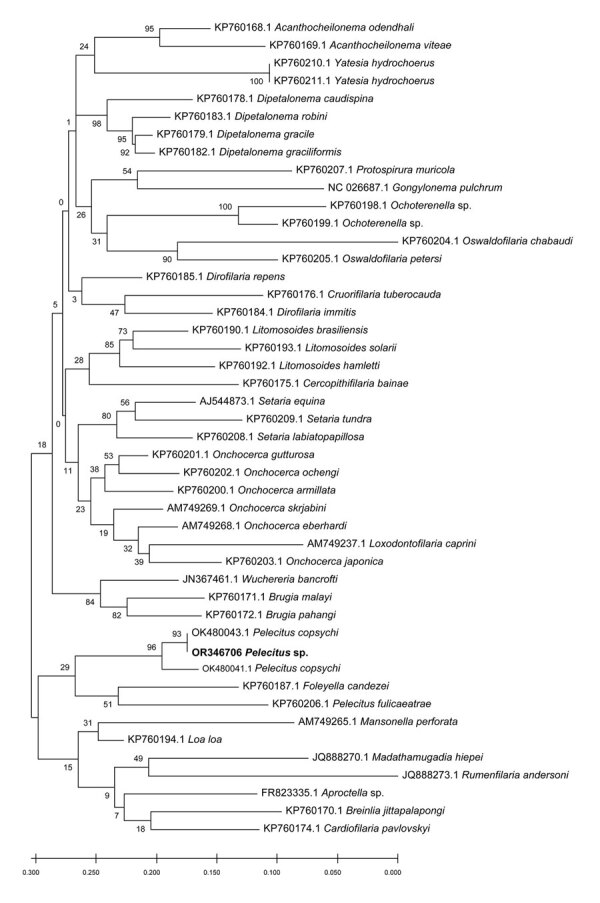
Maximum-likelihood reconstruction of phylogeny on the basis of *cox*1 sequences of *Pelecitus* sp. nematode isolated from the left eye of a 61-year-old man in Thailand (bold text) and reference sequences from GenBank. Bootstrap scores (percentages of 1,000 replications) are presented for each node. GenBank accession numbers are shown. Scale bar refers to a phylogenetic distance of 0.05 nucleotide substitutions per site.

Based on the best match in BLAST, the 12S rDNA sequence (468 bp) (Figure 3) showed 95%–96% identity with *P. copsychi* (GenBank accession nos. OK480976 and OK480977) and the *cox*1 sequence (611 bp) (Figure 4) showed 92%–96% identity with *P. copsychi* (GenBank accession nos. OK480041 and OK480043). The calculated p-distance for *cox*1 sequences between this causative agent and *P. copsychi* was 6.71%. We submitted sequences generated in this study to GenBank (accession nos. OR346706 [*cox*1] and OR396900 [12S rRNA]). 

## Discussion

The parasite specimen removed from this patient had a coiled body, rounded anterior and posterior extremities, distinct preesophageal cuticular ring, lateral alae, and a postdeirid. In addition, the vulva opened in the esophageal region. Those morphologic characteristics, along with the described morphometrics, indicated that the causative agent was a young, unmated female nematode of the genus *Pelecitus* (*5*,*18*). We compared that specimen with *P. copsychi*, *P. ceylonensis*, and *P. galli* nematodes from avian hosts in the Indomalayan realm (*5*,*7*,*8*). The specimen from our patient showed greater similarity to *P. copsychi* nematode than to the other 2 species, particularly in terms of body length. However, the esophagus of the specimen from our patient was shorter, only half the length of the *P. copsychi* esophagus. Although the morphometrics of microfilariae would have been necessary to differentiate species, the female nematode harbored no microfilariae. Nevertheless, we inferred that the taxonomic species of the specimen from our patient differed from *P. copsychi*. 

Our molecular analyses positioned the specimen from our patient near *P. copsychi* taxonomically (Figures 3, 4). The calculated p-distance for *cox*1 gene sequences between the specimen from our patient and *P. copsychi* was 6.71%. Filarial nematodes can be considered of the same species if the genetic distance based on their *cox*1 sequences is <2% (*19*). Interspecific distances are >4.8% in filariae. A 2017 study confirmed this distance threshold in the *Onchocerca* species (*20*). Therefore, genetic distances suggested the specimen from our patient differed from *P. copsychi* at the species level. However, because the specimen from our patient possessed the general morphologic characteristics of *Pelecitus* nematodes and was positioned near *P. copsychi* in the phylogenetic trees, we concluded that it is a congener, currently identified only as *Pelecitus* sp. 

Previously, 2 cases of zoonotic ocular infections possibly caused by *Pelecitus* spp. nematodes have been described in humans (*9*,*10*). The specimen from our patient was similar to the *Pelecitus* sp. nematode isolated from the iris fibers of a man in Brazil (*10*). The specimen from Brazil was a male worm with a preesophageal cuticular ring, lateral alae, and a postdeirid. In another case in Colombia, a male specimen of the genus *Loaina* was observed in the anterior chamber of the patient’s eye (*9*) but was later suggested to be of the genus *Pelecitus* (*4*,*10*). The study from Brazil (*10*) also stated that 2 specimens from humans in Colombia and Brazil were *Pelecitus* nematodes from birds. However, in the previous reports, no DNA sequences were obtained (*9*,*10*). Hence, we were unable to compare the sequences from our study with species from previous human ocular *Pelecitus* infections. 

Several modalities in the treatment of ocular parasitic infections have been described elsewhere. Lasers, including argon, Nd:YAG, and diode, are recommended to immobilize and kill the parasite before removal (*21*), but surgical removal is the mainstay of treatment options (*2*,*3*). Data are lacking regarding the efficacy of available anthelmintics for the treatment of *Pelecitus* infections (*22*). In animal studies, use of ivermectin, either alone or in combination with systemic steroids, may be effective against *Pelecitus* infection in macaws (*23*). In our case-patient, the intracameral parasite was successfully removed through surgery followed by treatment of the patient with postoperative topical antimicrobials and steroids. No recurrence occurred during the 1-year follow-up period. 

In conclusion, we report a case of human intraocular infection caused by a *Pelecitus* sp. nematode in Thailand. This finding expands the known geographic range of human infection with this zoonotic nematode, formerly reported only in South America. Guided by an initial morphologic analysis, molecular methods such as PCR can be useful for identifying rare infections such as *Pelecitus* sp. nematodes in humans, offering a rapid and accurate diagnostic approach. Healthcare providers should consider *Pelecitus* spp. nematodes as a possible causative agent in cases of small-to-moderate–sized helminths lodged in iris tissue. 
